# Effect of solvents extraction on phytochemical components and biological activities of Tunisian date seeds (var. Korkobbi and Arechti)

**DOI:** 10.1186/s12906-017-1751-y

**Published:** 2017-05-04

**Authors:** Amira Thouri, Hassiba Chahdoura, Amira El Arem, Amel Omri Hichri, Rihab Ben Hassin, Lotfi Achour

**Affiliations:** 1 Laboratoire de Recherche “Bioressourses, Biologie Integrative and Valorisation”, Institut Superieur de Biotechnologie de Monastir, Avenue Tahar Hadded, 74, 5000 Monastir, BP Tunisia; 2 Laboratoire des maladies transmissibles et substances biologiquement actives – Faculte de Pharmacie, Rue Avicenne, 5000 Monastir, Tunisia

**Keywords:** Polyphenols, Date seeds, Antioxidant activity, Antiinflamatory activity, α-amylase and α-glycosidase

## Abstract

**Background:**

The interest in natural antioxidants, especially polyphenols, is growing more and more thanks to their positive contribution to human health. Thus, the prevention from the harmful action of oxidative stress which has been involved in many diseases such as cancer, inflammation diabetes, and cardiovascular illness.

Recent research proved the bioactive compounds richness of date seeds which could be a good biological matrix of natural antioxidants. Unfortunately, an important quantity of Tunisian dates seed is discarded yearly.

**Methods:**

In this study, different solvents extraction (water, methanol, absolute acetone and aqueous acetone 80%) were used and the evaluation of its effect on phytochemical level, in vitro antioxidant activities, in vitro hyperglycemia key enzymes inhibition and in vivo anti-inflammatory proprieties were established for Tunisian date seeds.

**Results:**

The result revealed that the polar solvent exhibited the highest amount of bioactive compounds. The correlation between polyphenol compounds and the antioxidant potentiality explains the powerful effect of used polar solvents on inflammation, TBARS and hyperglycemia inhibition. Furthermore, it showed its higher capacity to scavenge radicals.

**Conclusions:**

Therefore, this big waste of Tunisian seeds could be used as cheap source of natural antioxidant compounds which are considered as a health challenge for the poor countries.

**Electronic supplementary material:**

The online version of this article (doi:10.1186/s12906-017-1751-y) contains supplementary material, which is available to authorized users.

## Background

The interest in natural antioxidants, especially polyphenols, is growing more and more thanks to their positive contribution to human health.

Several authors have proved the second metabolite richness of seeds in comparison to the edible portion of fruits [1, 2]. Unfortunately, an important quantity of Tunisian dates seed is discarded yearly as a result of food processes or used as animals food. This big waste could be used as a biological matrix and a cheap source of natural antioxidant compounds which are considered as a health challenge for the poor countries.

No toxicity is expected in seed extract and the previous studies reported no adverse effects of date pits on organ function, lipid profile, protein metabolism, hematological parameters, and body weight [3].

There is no doubt that oxidative stress has been involved in the initiation and/or the aggravation of many diseases such as Diabetes Mellitus (DM). It is a metabolic disorder characterized by the lost of blood glucose level’s control which increased as a result of the deficiency of insulin secretion or insulin effect [4]. It has been confirmed that the inhibition of carbohydrate hydrolyzing key enzymes such as α-amylase and α-glycosidase is an interesting approach in the postprandial blood glucose level control [5]. A range wide of side effects such as diarrhea, flatulence and abdominal bloating, are associated with the conventional inhibitors of those enzymes [6]. However, effects similar to insulin are showed when using of plant rich in polyphenols [7, 8].

Oxygen free radicals have been also reported to be involved in the enhancement of the inflammatory response and the affection of distant organs [9]. Furthermore, a wide range of toxic oxidative reactions is caused in the cell starting by the initiation of lipid peroxidation which leads to direct dysfunction of mitochondria, the random fragmentation of DNA and denaturation of enzymes [10]. The secondary metabolites of plants such as phenolics and flavonoids showed an excellent inhibition effect on inflammation as it acts as a suppressor of NF-Kβ [11].

Several reports focused on the anti-inflammatory activity of date fruits such as the work of Kehili et al. [12] However, few research works showed the inflammatory inhibition of the other *Phoenix dactylifera* parts. Although, the palm seeds has been used in the folk medicine as remedy and applied to wounds, lesions, inflammation, as we know, there are only the studies of Mohamed et Al-Okbi [13] and Arzi et al. [14] who proved the anti-inflammatory effect of date seed methanolic extract on adjuvant arthritis in rats as a model of chronic inflammation and on Carrageenan-Induced Inflammation in Male rat’s Hind Paw respectively.

Rodent models are widely used in inflammatory investigation experiments for its several useful features. It is considered superior to in vitro studies, simple and reliable. Furthermore, those findings can be projected to humans thanks to the close similarity of the overall physiological, molecular, and inflammatory response in rats and humans [15].

The reach and extraction of the secondary metabolites from plant material are a major focus of investigation. The presence of various phenolic families with different chemical structure and polarities results in the use of wide range of extraction solvents (water, acetone, methanol, ethanol, or their mixtures with water). However, despite the several works interests in the polyphenols extraction, there is no single solvent which may be considered standard because it is usually different for different plant matrices [16].

The objective of the study is determining the date seeds phytochemical composition and the effect of its different solvents extraction on its antioxidant activity, in vitro antidiabetic and in vivo anti-inflammatory proprieties in order to scientifically prove those biological activities of date seeds and find the best way to benefit its health power.

## Methods

### Samples

Two cultivars of date palm (*P. dactylifera* L.) fruits, Korkobbi and Arechti, were purchased from Gabes littoral oasis (Southern Tunisia), during the 2013 harvest season, at besser stage. The two varieties are authenticated by local farmers, and this authentication is confirmed by Rhouma Abdelmajid, National Coordinator in Tunisia and the voucher specimens were preserved with the code N° 20.8 for Korkobbi and N° 5.7 for Arechti in the National Institute of Agronomic Research of Tunisia (INRAT). The seeds, after been washed and air dried, was put at 50 °C [17] and ground into fine powder.

### Phytochemical determination

Each sample (1 g) was extracted twice by stirring with 30 mL of each solvent (methanol, absolute acetone and aqueous acetone), for the aqueous extract a decoction is prepared by boiling 100 g of seeds powder in 1 L of distilled water for 15 min, then the mixture was filtered and was dried at 40 °C. Each extract was redissolved in its appropriate solvent (final concentration, 5 mg/mL) for antioxidant activity evaluation.

The total phenolic content (TPC) were determied using a colorimetric assay described by Paras and Hardeep [18] and Reis et al. [19] based on the reduction of the Folin Ciocalteu reagent by the samples and expressed as mg of gallic acid equivalents (GAE) per g of extract.

For total flavonoid content (TFC), each extract (250 μl) was mixed with 1.25 ml of distilled water and 75 μl of 5% NaNO_2_ solution. After 5 min, 150 μl of 10% AlCl_3_ • H2O solution was added. After 6 min, 500 μl of 1 M NaOH and 275 μl of distilled water were added to prepare the mixture. The absorbance was read at 510 nm.

For the condensed tannins content (CTC), 50 μL of each extract was mixed with 1.5 mL of 4% vanillin and 750 μL of concentrated HCl. The solution was incubated for 20 min. The absorbance against blank was read at 500 nm [20].

(+)-Catechin was used as standard and the results were expressed as mg of (+)-catechin equivalents (CE) per g of extract.

### Antioxidant activity

Various concentrations of each date pits extract (0.3 ml) were mixed with 2.7 ml of methanolic solution containing DPPH radicals (6 × 10^−5^ mol/l). The antioxidant activity (AA) was measured using an improved ABTS method. 3.9 mL of ABTS^•+^ solution was added to 0.1 mL of the test sample and mixed vigorously, incubated for 6 min and read the absorbance at 734 nm.

For the reducing power, the test samples were mixed with sodium phosphate buffer (pH 6.6) and 1% potassium ferricyanide. After incubation at 50 °C for 20 min, 10% trichloroacetic acid was added and the mixture was centrifuged. 5 ml was mixed with deionised water and 0.1% ferric chloride, and read at 700 nm.

The extract concentration providing 0.5 of absorbance (EC50) was calculated from the graph of absorbance against extract concentration. Trolox was used as standard [21].

The thiobarbituric acid-reactive species (TBARS) assay was used to measure the amount of secondary product of the oxidation of polyunsaturated fatty acids called Malondialdehyde (MDA), formed from egg-yolk homogenates as lipid-rich matrix in a Fe ^2+^/ascorbate free-radical-induction system, in the presence and absence of various concentrations of each seeds extract. The resulting pinkish red chromogen was read with an absorbance maximum at 532 nm [22].

### In vitro diabetes key enzymes inhibition

#### α-Glucosidase inhibition assay.

The α-Glucosidase inhibitory activity of the both different seeds extracts (water, methanol, absolute acetone ad aqueous acetone) was determined according to the method of Tao et al. [23] with some modifications as reported by Rengasamy et al. [24]. A mixture of 2.5 mM p-nitrophenyl-α-glucopyranoside (pNPG), 250 μl of each extract (10–0.32 mg/ml) in DMSO and 0.3 U/ml of α-glucosidase in phosphate buffer, pH 6.9 was made. Control tubes contained only DMSO, enzyme and substrate. While in the positive control, Acarbose replaced the plant extracts. The inhibition capacity of extracts and Acarbose were calculated as following: Inhibition Percentage (%) = 1 − DO sample /DO control × 100. The result is expressed as IC50 (mg/ml) which revealed the inhibition concentration of each extract of 50% of intestinal α-glucosidase. All tests were carried out for three sample replications.

#### α-Amylase inhibition assay.

The α-amylase inhibition assay of the both different seeds extracts (water, methanol, absolute acetone and aqueous acetone) with varying concentrations from 0.16 to 10 mg/ml. was determined to base on the method described by Deguchi et al. [25] with slight modifications. The reaction mixture contained 500 μl of 1% starch solution, 400 μl of 0.1 M sodium phosphate buffer (pH 7.0), 50 μl of each sample extract dissolved in DMSO and 50 μl of pancreatic α-amylase (Sigma, St. Louis, USA) solution (2 U/ml). After the incubated at 37 °C for 10 min of the reaction medium, 3 ml of 3,5-dinitrosalicylic acid (DNS) color reagent was added. Finally, the solution was diluted with 20 ml of distilled water after its incubation in a boiling water bath for 5 min, and the absorbance was measured at 540 nm. The absorbance of a control sample was prepared accordingly without plant extract and acted as a negative control. The Acarbose was used as positive control. The inhibition capacity of extracts and Acarbose were calculated as following:

$$ \mathrm{Inhibition}\ \mathrm{Percentage}\ \left(\%\right)=1-\mathrm{DO}\ \mathrm{sample}/\mathrm{DO}\ \mathrm{control}\times 100. $$

The result is expressed as IC50 (mg/ml) which revealed the inhibition concentration of each extract and acarbose of 50% of pancreatic α-amylase. All tests were carried out for three sample replications.

### In vivo anti-inflammatory activity

Male Wistar rats (180–200 g, aged 12–14 weeks), obtained from Central Pharmacy of Tunis, Tunisia, were kept under standard laboratory conditions as acclimatization period for 2 weeks (23 °C, 55 ± 5% humidity and a 12 h light/dark cycle), and maintained with free access to water and a standard diet ad libitum. The animals were housed in groups of four in 595 × 380 × 200 mm cages and were monitored twice daily for health and clean behavior. No adverse events were observed. All animals were used according to the guidelines of the Tunisian Society for the Care and Use of Laboratory Animals, and the study was approved by the University of Tunisia Ethical Committee (approval number: FST/LNFP/Pro 152,012). All sections of this report adhere to the ARRIVE Guidelines for reporting animal research [26] (Additional file 1). A completed ARRIVE guidelines checklist is included in Checklist S1.

The anti-inflammatory activity was performed on the basis of the method of Winter et al. [27]. It consists in the inhibition of carrageenan (an edematogenic agent) - induced paw edema. Male Wistar rats were divided into different groups of 6 animals. The control group received 2.5 ml/kg of saline, the standard group received the reference drug (acetyl salicylate of lysine (ASL), 300 mg/kg) and the test groups received different date’ seeds extracts at a dose of 100, 200 and 300 mg/kg. Thirteen minutes later, 0.05 ml of 1% of carrageenan suspension was injected to all animals in the right hind paw. The paw volume, up to tibiotarsal articulation, was measured using a plethysmometer. The measures were determined at 0 h and 1, 3, and 5 h later.

The percentage of inhibition of each seeds extracts was determined as following:

$$ \%\mathrm{inhibition}=\left(\left(\mathrm{VT}-\mathrm{V}0\right)\ \mathrm{control}-\left(\mathrm{VT}-\mathrm{V}0\right)\ \mathrm{treated}\ \mathrm{group}\right)\times 100/\left(\mathrm{VT}-\mathrm{V}0\right)\ \mathrm{control}. $$

Where:

V0: the paw volume before edematogenic agent injection.

VT: the paw volume at 1, 3 and 5 h after edematogenic agent injection.

(VT − V0): was taken as the edema value.

### Statistical analysis

Results were given as mean ± standard deviation of 3 replicates. The results are expressed as mean values and standard deviation (SD). The results were analyzed using one-way analysis of variance (ANOVA) followed by Tukey’s HSD test with α = 0.05.

This treatment was carried out using SPSS 18.0 (SPSS Inc., Chicago, IL, USA) software.

Data obtained from animal experiments were expressed as means ± SE and as percentage. Results were statistically evaluated by ANOVA and using Student’s t-test. *p* ≤ 0.05 were considered significant.

## Results and discussion

### Solvents extraction effect on phytochemical composition of date pits

**Fig. 1:**
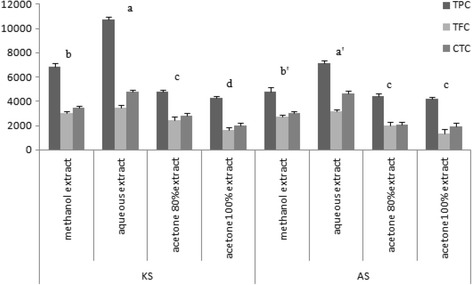
Effect of different solvents on phytochemical composition of both date seeds. Data expressed as means ± standard deviations of three independent extractions (*n* = 3). TPC: Total phenolic content (mg GAE per 100 g of extract), TFC: Total flavonoid content (mg CE per 100 g of extract), CTC: Condensed tannins contents (mg CE per 100 g of extract), Different lower case letters in the same types of material indicate significant differences between the different used solvents extraction (*P* < 0.05). KS: Korkobbi seed, AS Arechti seed

The total phenolic, flavonoids and condensed tannins of water, methanol, absolute acetone and aqueous acetone of both seeds extract are depicted in Fig. 1.

It was elucidated that the water and methanol extracts showed higher phenolic content than the acetones extract. Water and methanol seed extract also exhibited a high content of flavonoid and condensed tannins (Fig. 1).The total polyphenolic content with regards to different solvents used for extraction was as follows: water > methanol > aqueous acetone > absolute acetone.

A good solvent is characterized by its optimal extraction and its capacity in conserving the stability of the chemical structure of desired compounds [28].

Therefore the type of extraction solvent and its polarity may have a significant impact on the level of extracted polyphenols. The polarities of the polyphenols range from polar to non-polar, optimum extraction of polyphenols is usually obtained in the polar solvent which have a better efficiency of solvation as a result of interactions (hydrogen bonds) between the polar sites of the antioxidant compounds and the solvent than nonpolar one [29]. Therefore, water and an aqueous mixture of methanol and ethanol are frequently used for recovering polyphenols. Acetone gave a low level of antioxidant compounds because of their lower efficiency of solvation. The acetone molecules are known as proton acceptors only while methanol and water, are also proton donors.

### Solvents extraction effect on antioxidant activities of date pits

The antioxidant activities of water, methanol, absolute acetone and aqueous acetone of both seeds extract against DPPH, ABTS, FRAP and TBARS are depicted in Table 1.

**Table 1:** Effect of solvents extraction on antioxidant activity (IC_50_. mg mL^−1^) of both date palm seeds

Extracts		IC 50			
		DPPH	ABTS	FRAP	TBARS
KS	Methanol extract	0,74 ± 0,02^b^	0,40 ± 0,09^a^	0,27 ± 0,01^b^	0,42 ± 0,02^a^
	Aqueous extract	0,35 ± 0,01^a^	0,37 ± 0,03^a^	0,12 ± 0,01^a^	0,32 ± 0,02^a^
	Acetone 80%	1,41 ± 0,81^c^	0,82 ± 0,04^b^	0,46 ± 0,03^c^	0,75 ± 0,09^b^
	Acetone 100%	1,88 ± 0,67^d^	1,13 ± 0,34^c^	0,73 ± 0,045^d^	0,99 ± 0,07^c^
AS	Methanol extract	0,61 ± 0,04^B^	0,53 ± 0,05^A^	0,26 ± 0,01^A^	0,47 ± 0,09^A^
	Aqueous extract	0,58 ± 0,09^A^	0,48 ± 0,07^A^	0,19 ± 0,01^A^	0,42 ± 0,05^A^
	Acetone 80%	1,61 ± 0,53^C^	1,19 ± 0,98^B^	0,71 ± 0,08^B^	0,98 ± 0,09^B^
	Acetone 100%	2,01 ± 0,98^D^	1,40 ± 0,65^B^	0,86 ± 0,04^B^	1,22 ± 0,07^C^

Data expressed as means ± standard deviations of three independent extractions (*n* = 3)

IC_50_: The concentration at which 50% of radicals are scavenged, Fe3+ is reduced and TBARS is inhibited

Different lower case letters and capital letters in the same column for each type of material indicate significant effect differences between the different used solvents extraction (*P* < 0.05)

*KS* Korkobbi seed, *AS* Arechti seed

The IC50 of the extract is inversely related to its antioxidant compounds richness (Lower IC50 values indicate a higher antioxidant activity).

It expresses the amount of antioxidant required to decrease the DPPH and the ABTS concentration by 50%, to reduce the 50% of Fe_3_^+^/ferricyanide complex to the Fe_2_^+^ and to inhibit the 50% of lipid peroxidation revealed by the decrease of the formation of MDA as its resulted product.

It was elucidated that the water and methanol extracts showed higher antioxidant activities than the acetones extract. The antioxidant activities with regards to different used solvents were as follows: water > methanol > aqueous acetone > absolute acetone.

There is a wide range of plant phytochemical components. Among them, phenolic compounds lie on the head of several research interests. This importance due mainly to the polyphenol capacity in preventing many diseases at the bottom of which lies the reactive species of oxygens such as cancer, inflammation, diabetes, cardiovascular disease etc... . It includes phenolic acids, hydrolyzable and condensed tannins, and flavonoids. The efficiency of these compounds is explained by their antioxidant action as free radical scavengers, hydrogen donors and reducing agents [30].

The antioxidant power of the samples is closely associated with their total phenolic content (Table 2).

**Table 2:** Pearson’ correlation analysis between the antioxidant activity and phytochemical composition of both common date palm seeds

	TPC	TFC	CTC
DPPH	-0.987**	−0.832**	-0.794**
ABTS	-0.982**	−0.964**	−0.909**
Reducing Power	−0.983**	−0.938**	-0.901**
TBARS inhibition	−0.953**	−0.949**	−0.951**

**Significant correlation for *p* < 0.01

*TPC* Total phenolic content, *TFC* Total flavonoid content, *CTC* Condensed tannins content. *TAC* Total anthocyanins content

The extracts with higher levels of total phenolics, also exhibit greater antioxidant activity. It is concluded that the change in solvent polarity influences the dissolution of selected antioxidant compounds and the antioxidant activity estimation. Change in solvent polarity alters its ability to dissolve a selected group of antioxidant compounds and influences the antioxidant activity estimation [31].

### Solvents extraction effect on in vitro hyperglycemia key enzymes inhibition of date pits

**Fig. 2:**
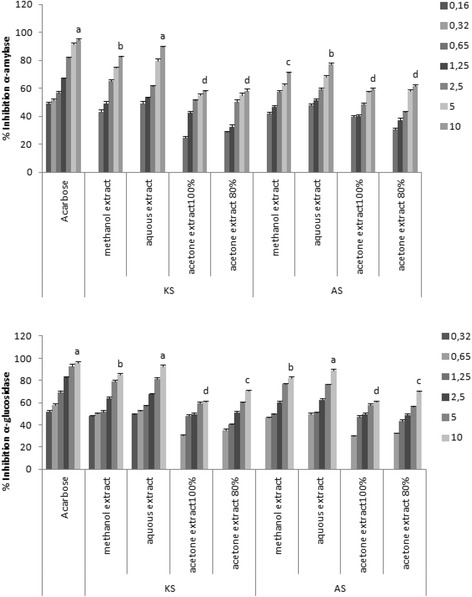
Effect of different solvents on hyperglycemia key enzymes inhibition of both date seeds. Different concentrations (0.32–10 mg mL^−1^) from each seed extract are used. Data expressed as means ± standard deviations of percent enzyme activity. Different lower case letters in the same types of material indicate significant differences between the different used solvents extraction (*P* < 0.05). KS: Korkobbi seed, AS Arechti seed

Four extracts of two varieties *Phoenix dactylifera* seeds Arechti and Korkobbi ones were evaluated for their in vitro inhibitory effect on α-amylase and α-glucosidase enzymes (Fig. 2). The four extracts (methanolic, aqueous, and acetone at two concentration 100% and 80%) of Korkobbi seed (at a concentration of 10 mg/ml) exhibited 82.28, 89.45, 57.8 and 58.16 α-amylase inhibitory activity and 84.99, 92.39, 60.48 and 70.62 α-glucosidase inhibitory activity, respectively. Whereas, for Arechti seed, the same extracts exhibited 71.07, 77.25, 59.03 and 61.8 α-amylase inhibitory activity and 81.98, 88.68, 60.58 and 69.83 α-glucosidase inhibitory activity, respectively.

Acarbose was used as a standard reference drug, which showed α-amylase inhibitory activity with an IC50 value of 0.232 mg/ml and α-glucosidase inhibitory activity with an IC50 value of 0.161 mg/ml. Among all, aqueous extract of Arechti seed and Korkobbi seed has shown best enzyme inhibitory activity with an IC50 value 0.783 and 0.353 mg/ml (α-amylase and α-glucosidase) and 0.987 and 0.768 mg/ml (α-amylase and α-glucosidase) which were comparable with that of acarbose in comparison with other extracts (Table 3).

**Table 3:** IC50 of α-amylase and α-glucosidase inhibition of different seed extracts

Samples		IC50 (mg/ml)	
		α-amylase	α-glucosidase
Acarbose		0,23 ± 0,01	0,16 ± 0,02
KS	Methanol extract	1,31 ± 0,08*	0,65 ± 0,05
	Aqueous extract	0,78 ± 0,05	0,35 ± 0,09
	Acetone 80% extract	2,33 ± 0,6**	2,56 ± 0,9**
	Acetone100% extract	2,47 ± 0,8**	2,40 ± 0,79**
AS	Methanol extract	1,65 ± 0,1*	1,31 ± 0,8*
	Aqueous extract	0,99 ± 0,2*	0,77 ± 0,05
	Acetone 80% extract	2,88 ± 0,83**	2,72 ± 0,78**
	Acetone100% extract	3,66 ± 0,99**	2,85 ± 0,86**

Data expressed as means ± standard deviations of three independent extractions (*n* = 3)

IC_50_: the concentration at which 50% of enzyme is inhibited

*KS* Korkobbi seed, *AS* Arechti seed

*Significant difference for *P* < 0.05 and **Significant difference for *P* < 0.01

Interestingly, there was a dose-dependent increase in percentage inhibitory activity against alpha amylase and alpha-glucosidase enzymes. The aqueous extract of Korkobbi seed showed marginally more inhibitory effect, which could be explained by its more detected polyphenols compounds.

Diabetes mellitus is a chronic metabolic disorder due to an ineffective use of insulin and it is identified by hyperglycemia which is a classical risk associated with a rise of reactive oxygen species production, leading to oxidative tissue damage and diabetic complications [32].

One of the useful strategies for treatment of diabetes is the control of post-prandial hyperglycemia. This can be accomplished by the inhibition of the carbohydrate hydrolyzing enzymes α-amylase which is in charge of the breakdown of 1, 4-glycosidic linkages of polysaccharides (starch, glycogen) to disaccharides and α-glucosidase which catalyzes the disaccharides to monosaccharides. Inhibitors of these enzymes delay carbohydrate digestion and reduce the rate of glucose retention which consequently diminish the postprandial plasma glucose rise [33–35]. Synthetic inhibitor such acarbose, miglitol and voglibose are reported to cause an adverse effect such as different gastrointestinal symptoms including abdominal pain, diarrhea [36]. Hence, herbal medicines are getting more importance as natural carbohydrate hydrolyzing enzyme inhibitors having fewer/or no side effects [37].

Many bioactive compounds from plants, in that mostly phenolics, triterpenoids and flavonoids have a positive correlation as antidiabetic agents [38–40].

Polyphenolic compounds may reduce the potency of α-amylase and α-glucosidase by the interaction or inhibition of specific positions in enzymes [41].

The flavonoids are known as a puissant blocker of glucose absorption and inhibitor of sodium-dependent glucose transporter-1 improving glucose tolerance.

Furthermore, some flavonoid compounds like luteolin and kaempferol may inhibit the α-amylase and α-glucosidase activity in the intestine [42, 43].

In correlation with previous reports, several results revealed the in vitro inhibition activity of carbohydrate hydrolyzing enzymes of *Phoenix dactylifera* fruits and seeds thanks to their richness with secondary metablites such as polyphenols, flavonoids and tannins [44, 45].

### Solvents extraction effect on in vivo anti-inflammatory activity of date pits

The anti-inflammatory activity of four extracts of the two *Phoenix dactylifera* varieties seeds Arechti and Korkobbi were evaluated using the carrageenan-induced rat paw edema model (Table 4: at bottom). When injected locally into the sub plantar region of rat paw of the control group, carrageenan induced a severe inflammatory reaction remained even 6 h after its injection. The presence of edema is one of the prime signs of inflammation. The maximum peak was observed between 3 and 5 h after injection. Intraperitoneal injection of seeds extracts produced a significant reduction of edema in a dose-related manner. Interestingly, the highest reduction of the edema of the extracts was at 3 h. The aqueous extract of both varieties seeds showed the highest inhibitory edema formation, followed by the methanolic one then the acetone extracts, marked especially at 300 mg/kg with which the edema was found to be reduced to the extent of 66.90% and 63.52% for Korkobbi and Arechti seeds respectively. This value is comparable to that of the reference drug, ASL, a potent inhibitor of cyclooxygenase-2 (300 mg/kg) which decreased paw edema by 57.42% at the third hour.

**Table 4:** Effect of solvents extraction on in vivo anti-inflammatory activity of both date seeds

			Edema (10–^2^/mL) (mean ± SD)			Edema inhibition (%)		
Treatment		Dose (mg/kg)	1 h	3 h	5 h	1 h	3 h	5 h
Control			34 ± 1,5	59,5 ± 1,7	68,3 ± 1,09			
KS	Methanol extract	100	27,03 ± 1,9	29,26 ± 1,1*	35,01 ± 0,89*	20,5	50,82	48,74
		200	23,08 ± 2,8*	24,11 ± 0,8*	30,13 ± 1,04*	32,11	59,47	55,88
		300	19,1 ± 1,5**	23,34 ± 1,8**	28,04 ± 1,65*	43,82	60,77	58,94
	Aquous extract	100	25,5 ± 1,07	27,12 ± 2,02*	33,37 ± 1,98*	25	54,42	51,14
		200	20,17 ± 2,3*	22,02 ± 1,98**	27,52 ± 2,67*	40,67	62,99	59,70
		300	17 ± 2,7**	19,69 ± 1,08**	24,6 ± 2,13*	50	66,90	63,98
	Acetone extract 80%	100	29,24 ± 2,1	35,17 ± 2,09*	38,14 ± 2,87*	14	40,89	44,15
		200	26,18 ± 1,03	29,04 ± 1,98*	34,21 ± 1,89*	23	51,19	49,91
		300	21,41 ± 1,75*	26,3 ± 1,91*	32,12 ± 2,09*	37,02	55,79	52,97
	Acetone extract 100%	100	30,09 ± 0,89	37,15 ± 2,09	41,07 ± 2,01*	11,5	37,56	39,86
		200	27,23 ± 2,87	32,21 ± 2,02*	39,51 ± 1,09*	19,91	45,86	42,15
		300	23,35 ± 1,98*	27,5 ± 1,89*	34,21 ± 1,19*	31,32	53,78	49,91
AS	Methanol extract	100	27,5 ± 1,76	31,15 ± 1,09*	36,29 ± 1,2*	19,11	47,64	46,86
		200	24,41 ± 1,57	27,21 ± 2,08*	32,14 ± 1,14*	28,20	54,26	52,94
		300	20,06 ± 1,4*	24,5 ± 2**	30,34 ± 2,03*	41	58,82	55,57
	Aquous extract	100	26,18 ± 2,34	28,41 ± 2,02	34,21 ± 3,01*	23	52,25	49,91
		200	21,5 ± 2,04*	23,34 ± 1,03*	29,02 ± 1,2*	36,76	60,77	57,51
		300	18,05 ± 2,01**	21,7 ± 1,65*	27 ± 2,98*	46,91	63,52	60,46
	Acetone extract 80%	100	28,5 ± 1,87	35,17 ± 1,05	38,04 ± 2,07*	16,17	40,89	44,30
		200	26,18 ± 1,94	31,97 ± 2,3	35,15 ± 1,84*	23	46,26	48,53
		300	23,12 ± 1,87*	28,41 ± 2,03	34,21 ± 1,90*	32	52,25	49,91
	Acetone extract 100%	100	30,83 ± 1,02	38,38 ± 1,98	45,24 ± 2,03*	9,32	35,49	33,76
		200	29,7 ± 1,09	34,29 ± 1,25	41,17 ± 2,5*	12,64	42,36	39,72
		300	25,47 ± 2,01	28,17 ± 1,38	35,05 ± 1,76*	25,08	52,65	48,68
Asl		300	22,29 ± 1,09*	25,33 ± 1,34	31,61 ± 1,87	34,44	57,42	53,71

Values are expressed as mean ± S.E.M. (*N* = 6); ASL: acetyl salicylate of lysine

*KS* Korkobbi seed, *AS* Arechti seed

*p ≤ 0.05 significant from the control. **p ≤ 0.01 significant from the control. ***p ≤ 0.001 significant from the control

Although Inflammation is a defense mechanism which is involved in the injurious tissues healing process, the libiration of mediators responsible of protective reaction such as cytokine, histamine, serotonin, leukotrienes and prostaglandin can induce, maintain or aggravate many diseases such as atherosclerosis, rheumatoid arthritis, asthma, and neurodegenerative diseases [46].

Carrageenan-induced paw edema has been widely used as a useful phlogistic tool for investigating the anti-edematous effect of natural products. It is known as a biphasic agent, the early phase (90–180 min) of the inflammation is characterized by the release of histamine, serotonin and similar substances. The later phase (270–360 min) is known by the activation of kinin-like substances and the release of prostaglandins, proteases and lysosome [47, 48].

Any interruption of those phases allows the reduction of the liberation of the mediators then the establishment of normal state [49].

Many of the frequently used anti-inflammatory drugs are becoming less acceptable due to serious side effects on human health such as gastric intolerance, bone marrow depression and water and salt retention [50].

Several reports showed that free radicals are important mediators that induce or aggravate inflammatory processes and, consequently, antioxidants and radical scavengers can decrease inflammation [51, 52].

Previous studies have indicated that the major pharmacological activities of *Phoenix dactylifera* seeds are derived from the richness of those by product with the secondary metabolites such as polyphenols, flavonoids, tanins and terpenes.

The studied seeds extracts inhibited the both phases of the carrageenan-induced edema by reducing the release of histamine and serotonin and also the kinin-like substances and prostaglandins. This pharmacological property may be attributed to their chemical composition marked by different calasses of plyphenols, flavonoids and vitamin like tocopherol, cinamic acid.... [53].

## Conclusion

Extracting solvent significantly affected phytochemical content (total polyphenols, flavonoids, and condensed tannins), antioxidant activity (DPPH, ABTS, FRAP, and TBARS) and the inhibition capacity in both biological activities (Inflammation and hyperglycemia key enzymes) of date seeds extract. The most efficient solvents for polyphenols extraction were water and methanol. For both seeds extracts, polyphenol content of absolute acetone extracts was the lowest. The two polar solvents showed the highest antioxidant activity, a powerful inhibition against TBARS, carrageenan-induced edema and diabetes associated key enzymes.

A good correlation was obtained between antioxidant properties of date seed extracts. Therefore, the aqueous date seeds extract could be considered as a cheap and safe source of natural antioxidants and involved in different applications both in the field of the food industry and for the pharmacological use.

## Additional file

Additional file 1: The ARRIVE Guidelines Checklist. (PDF 1 kb)

## Abbreviations

ABTS: 2,2′-Azinobis-3-ethylbenzothiazoline-6-sulfonic acid
DPPH: 1,1-Diphenyl-2-picrylhydrazyl
TBARS: Thiobarbituric acid-reactive substances
